# Microbial Inhibition of *Fusarium* Pathogens and Biological Modification of Trichothecenes in Cereal Grains

**DOI:** 10.3390/toxins9120408

**Published:** 2017-12-20

**Authors:** Urszula Wachowska, Danuta Packa, Marian Wiwart

**Affiliations:** 1 Department of Entomology, Phytopathology and Molecular Diagnostics, University of Warmia and Mazury in Olsztyn, ul. Prawocheńskiego 17, 10-720 Olsztyn, Poland; urszula.wachowska@uwm.edu.pl; 2 Department of Plant Breeding and Seed Production, University of Warmia and Mazury in Olsztyn, pl. Łódzki 3, 10-724 Olsztyn, Poland; packa@uwm.edu.pl

**Keywords:** antagonistic bacteria, antagonistic fungi, biological control, biological modification of fusariotoxins, cereals

## Abstract

Fungi of the genus *Fusarium* infect cereal crops during the growing season and cause head blight and other diseases. Their toxic secondary metabolites (mycotoxins) contaminate grains. Several dozen toxic compounds produced by fungal pathogens have been identified to date. Type B trichothecenes—deoxynivalenol, its acetyl derivatives and nivalenol (produced mainly by *F. graminearum* and *F. culmorum*)—are most commonly detected in cereal grains. “T-2 toxin” (produced by, among others, *F. sporotrichioides*) belongs to type-A trichothecenes which are more toxic than other trichothecenes. Antagonistic bacteria and fungi can affect pathogens of the genus *Fusarium* via different modes of action: direct (mycoparasitism or hyperparasitism), mixed-path (antibiotic secretion, production of lytic enzymes) and indirect (induction of host defense responses). Microbial modification of trichothecenes involves acetylation, deacetylation, oxidation, de-epoxidation, and epimerization, and it lowers the pathogenic potential of fungi of the genus *Fusarium*. Other modifing mechanisms described in the paper involve the physical adsorption of mycotoxins in bacterial cells and the conjugation of mycotoxins to glucose and other compounds in plant and fungal cells. The development of several patents supports the commercialization and wider application of microorganisms biodegrading mycotoxins in grains and, consequently, in feed additives.

## 1. Introduction

In temperate climates, pathogens of the genus *Fusarium* are the most dangerous producers of toxic metabolites in cereal grains. They develop during the growing season on spikes or panicles to cause *Fusarium* head blight (FHB). A number of secondary metabolites produced by those fungi have an adverse influence on mammals: they cause damage to internal organs and lead to poisoning in humans and animals [1]. Fungi of the genus *Fusarium* are not always effectively controlled with fungicides because FHB epidemics develop rapidly and involve at least several fungal species [2]. New strategies in crop protection aim to limit the number of fungicide treatments, and they have led to the introduction of integrated pest management requirements in the relevant legal acts and regulations [3] which favor biological and biotechnological approaches. Statutory regulations significantly limit the use of chemical substances in organic farms [4]. The mycotoxin content of grains intended for consumption should not exceed the levels determined to be safe by toxicological considerations [5,6]. In unprocessed soft wheat grains, the maximum concentrations of deoxynivalenol (DON) are set at 1250 µg kg^−1^, in durum and oat grains at 1750 µg kg^−1^ [5], and the total content of T-2 and HT-2 toxins (indicative value) may not exceed 100 µg kg^−1^ [7]. Due to food safety concerns, mycotoxin levels in food and feed have to be monitored regularly to evaluate the risk of exceeding the tolerable daily intake (TDI) in humans and animals, expressed in mg kg^−1^ body weight (bw) [8]. Food and feed producers are, thus, forced to search for new methods of inhibiting the growth and development of toxin-producing fungi of the genus *Fusarium*.

Genetic analyses investigating the interactions between the genes of toxin-producing fungi, bacteria, antagonistic fungi and host plants supply valuable data for designing new biotechnological methods for eliminating trichothecenes from plant tissue. Okubara et al. [9] described a wheat line transformed by the *TRI 101* gene from *F. sporotrichioides*. *TRI101* encodes an enzyme that transfers an acetyl moiety to the C3 hydroxyl group of trichothecenes. Acetyltransferase in this plant confers partial protection against the spread of *F. graminearum* in inoculated wheat heads. Ohsato et al. [10] obtained resistant rice genotypes showing DON acetylase activity. Genetic transformation can significantly increase cereal resistance to *Fusarium* fungi [10]. However, this method is rarely used, and it is subject to numerous legal limitations in Europe. Genetic transformation complements the natural defense mechanism of cereals, which involves the conjugation of DON to glucose to produce (deoxynivalenol-3-β-d-glucopyranoside) D3G [11,12]. This mechanism probably differentiates the susceptibility of different wheat species to *F. culmorum* infections [13]. The presence of DON-glutathione conjugates was reported in wheat [14]. The cited authors also observed other conjugates, including DON-cysteine, as well as unknown conjugates formed in plants.

The microbiome of cereal grains, which is composed of various species of yeasts, bacteria, and filamentous fungi, is found on grain surfaces and in grain tissues, and it is much more abundant than toxin-producing fungi of the genus *Fusarium* [15,16]. The microbiome’s role in protecting plants against pathogens is intensively studied [15,17,18,19]. The microbiome can prevent the growth of *Fusarium* pathogens by antibiosis and competition during the growing season [20,21] and grain storage [22]. It also enhances host defense against pathogens [23,24]. Numerous studies have demonstrated that microorganisms can biotransform or biodegrade mycotoxins to compounds that are non-toxic or less toxic [25,26]. The aim of this review article was to summarize the existing knowledge regarding the use of selected microorganisms for inhibiting the development of *Fusarium* pathogens and lowering trichothecene concentrations in cereal grains and cereal-based feed.

## 2. Trichothecenes

Trichothecenes are one of the major groups of secondary metabolites produced by species of the genus *Fusarium* and covering more than 200 compounds [27,28]. The chemical structure and the possible biosynthesis pathways of trichothecenes are relatively well known [28]. Several questions regarding the toxicity mechanisms of trichothecenes remain unanswered, but some of these mechanisms have been identified in eukaryotes [29]. Trichothecenes are cyclic terpenoids which can be further subdivided into simple and macrocyclic forms. Simple trichothecenes are a family of tetracyclic or tricyclic sesquiterpenes with various substituents [28]. FHB is caused mainly by *F. graminearum* and *F. culmorum* which produce type B trichothecenes—DON with its acetylated derivatives, 3-acetyl-DON (3-Ac-DON) and 15-acetyl-DON (15-Ac-DON), and nivalenol (NIV). Fungi that are less frequently noted on cereal spikes include *F. sporotrichioides* and *F. poae* which produce type-A trichothecenes-T-2 toxin and HT-2 toxin [30].

Type-B trichothecenes suppress appetite, induce vomiting and weaken the immune system [27,31]. T-2 toxin and other type-A trichothecenes damage the skin and mucous membranes, promote vomiting and diarrhea, suppress appetite, lead to hemorrhaging and neurological disorders [32]. Trichothecenes often disrupt life processes in mammalian cells. These metabolites cause various human and animal diseases classified as mycotoxicoses. Goossens et al. [33] demonstrated that the intake of feed contaminated with 903 ± 85 μg kg^−1^ of T-2 toxin inhibited the activity of porcine liver enzymes within 14 days. At the cellular level, trichothecenes disrupt nucleic acid synthesis, mitochondrial function, membrane integrity, and cell division. Trichothecenes may also induce apoptosis in animal cells [29]. DON and T-2 toxin bind to the peptidyl transferase center of the ribosome and block protein synthesis [34].

The biosynthesis of trichothecenes begins with the cyclization of farnesyl pyrophosphate (FPP) to form trichodiene, which then undergoes a series of oxygenation, isomerization, cyclization, and esterification reactions to form T-2 toxin, NIV, or DON [35,36,37]. The transformation pathway was described in a previous study [38]. Proteins that participate in the biosynthesis of trichothecenes are encoded by *TRI1-TRI16* genes grouped in three clusters. *F. graminearum sensu stricto* and *F. sporotrichioides* were the first model species where the main *TRI* cluster was characterized [39,40,41]. In both species, the main *TRI* cluster is composed of 12 genes responsible for the synthesis and modification of the trichothecene skeleton. The cluster can include seven genes (*TRI8*, *TRI7*, *TRI3, TRI4*, *TRI5*, *TRI11*, *TRI13*) encoding enzymes that catalyze 10 trichothecene biosynthetic reactions, two genes (*TRI6* and *TRI10*) encoding transcriptional regulators, one gene (*TRI12*) encoding a transport protein, and two genes (*TRI9* and *TRI14*) with uncertain functions. Both species have two additional *TRI* loci: the single gene *TRI101* locus encoding C-3 acetyltransferase and a locus containing two genes—*TRI1* encoding C-8 oxygenase and *TRI16* encoding C-8 acetyltransferase. An analysis of the fungal genome revealed that those loci are positioned on different chromosomes [30,42]. The presence of the *TRI5* gene, which encodes the enzyme participating in the isomerization and cyclization of farnesyl pyrophosphate to trichodiene in *Fusarium* isolates, determines their ability to produce trichothecenes. The chemotypes of *Fusarium* isolates can be determined by analyzing the polymorphism of *TRI3*, *TRI7*, *TRI12*, and *TRI13* genes in the main *TRI* cluster and *TRI1* in additional *TRI* loci containing genes responsible for the synthesis of various types of trichothecenes. For example, the development of the NIV chemotype of *F. graminearum* requires the expression of *TRI7* and *TRI13* genes, whereas their absence leads to the development of the DON chemotype [41,43]. *TRI1* was verified to be responsible for the difference in hydroxylation at C-8 and allows to distinguish DON chemotypes from chemotypes defined as NX. The NX chemotypes due to the lack of a ketone group at C-8 are classified as type-A trichothecenes [44].

## 3. Inhibition of the Growth of *Fusarium* Pathogens and Trichothecene Production by Microorganisms

The above goal can be achieved through biological treatments where microorganisms are applied to plants during the growing season. Microorganisms adapt well to the surface of wheat spikes, they reduce symptoms of FHB and DON levels in kernels. Schisler et al. [20] demonstrated that the biocontrol agent *Cryptococcus flavescens* OH 182.9 delivers protective effects on wheat spikes (Table 1). DON can inhibit the activity of *N*-acetyl-beta-d-glucosaminidase, an enzyme which is produced by the antagonistic species *Trichoderma atroviride* and is capable of degrading the cell walls of pathogens [45]. Therefore, antagonistic microorganisms tolerant of the secondary metabolites produced by *Fusarium* pathogens have to be identified. In a study by Pan et al. [15] isolates of endophytic *Bacillus megaterium* (BM1) and *Bacillus subtilis* (BS43, BSM0, BSM2) bacteria inhibited the growth and spore formation in *F. graminearum,* thus decreasing DON levels in field-grown wheat by up to 89.3%. In an earlier study, Dunlap et al. [46] demonstrated that the above bacterial species produced fungicidal or fungistatic metabolites: surfactin, iturin and fengycin lipopeptides. Palazzini et al. [47] reported that antagonistic bacterium *Bacillus velezensis* RC 218 could significantly reduce FHB severity and DON accumulation under field conditions. Zhao et al. [48] demonstrated that a cell-free *Lactobacillus rhamnosus* culture supernatant can effectively inhibit in vitro growth of *F. culmorum*. Baffoni et al. [49] found that *Lactobacillus plantarum* isolated from silages and *Bacillus amyloliquefaciens* isolated from forest soil inhibited the growth of *F. culmorum* and *F. graminearum* in vitro. The cited authors also demonstrated that weekly biological treatments (applied from heading until anthesis) involving a mixture of two isolates of the above species were more effective in reducing the symptoms of FHB in *T. durum* than two treatments applied in the flowering stage.

**Figure 1:**
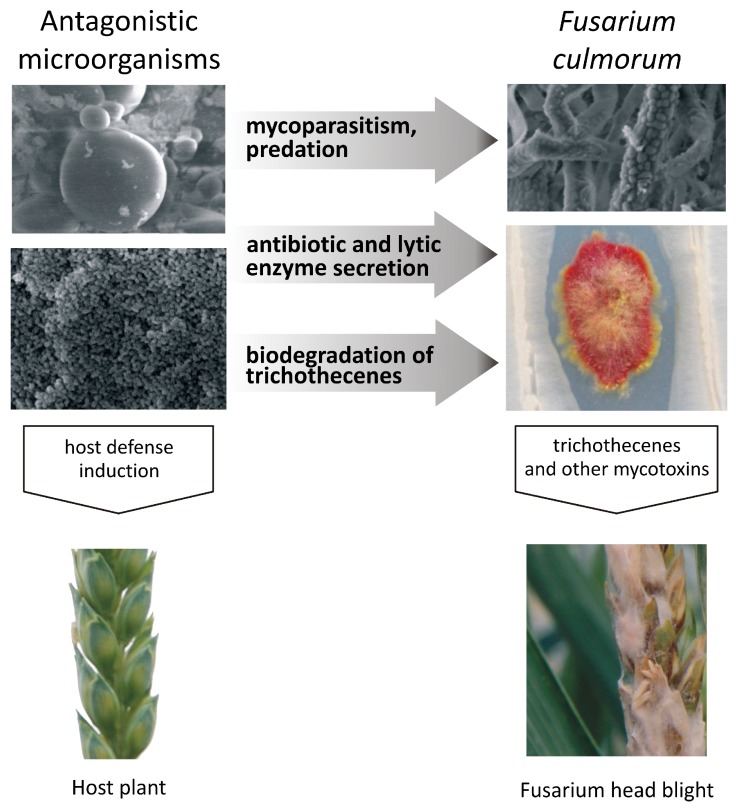
Inhibition of *Fusarium* pathogens by antagonistic microorganisms: direct inhibition (mycoparasitism, predation), mixed-path antagonism (antibiotic and lytic enzyme secretion, biological modification of trichothecenes), and indirect inhibition involving interactions with the host plant (induction of host defense responses).

Antagonistic bacteria and fungi can affect pathogens of the genus *Fusarium* via direct, mixed-path and indirect modes of action (Figure 1). Direct interactions include mycoparasitism and hyperparasitism (Table 1). They have been reported by Vujanovic and Goh [57] in a study evaluating the effectiveness of *Sphaerodes mycoparasitica* filamentous fungi against *F. graminearum.* The cited authors hypothesized that *S. mycoparasitica* absorbs aurofusarin from the attacked *Fusarium* cells by lysing the components of pathogenic cell membranes, such as chitin, and producing chitinase and chitosanase. Ferre and Santamarina [60] demonstrated that *Trichoderma harzianum* produces antifungal extracellular metabolites, including enzymes that degrade the wall of *F. culmorum* cells: chitinases, glucanases and proteases. The filamentous fungi *Clonostachys rosea* analyzed by Mamarabadi et al. [59] also produced enzymes that degrade cell membranes in *F. culmorum.*

Mixed-path antagonism involves antibiotic secretion and the production of volatile antagonistic compounds. Chan et al. [50] demonstrated that culture filtrates of *B. subtilis* effectively reduced the severity of FHB (*F. graminearum*) in wheat and lowered DON levels in grains by up to 51% relative to control. Culture filtrates contained metabolites of the analyzed bacterium. They were identified as fengycin-like isoforms which are members of the family of membrane-active lipopeptide antibiotics against filamentous fungi. Shi et al. [51] analyzed the effectiveness of *B. amyloliquefaciens* in reducing DON concentrations in wheat grains. The experiment was conducted in a controlled environment, and the growth of *F. graminearum* mycelia in wheat kernels was observed in flasks containing bacterial cell-free culture supernatant. In the work of Nagaraja et al. [52], zones of inhibition were observed on Petri plates where *Azotobacter nigricans* was cultured in the presence of several *Fusarium* species. According to the authors, the analyzed bacterial species can be effectively used to protect wheat, maize and sorghum against toxin-producing fungi of the genus *Fusarium*. The tested strain of *A. nigricans* also stimulated root and shoot growth in the analyzed plants. Bacteria of the genera *Bacillus*, *Pseudomonas* and *Microbacterium* from wheat and barley rhizosphere [53], as well as *B. cereus* isolated from wheat [54] produced volatile antagonistic compounds that inhibited the development of *F. culmorum* and *F. graminearum* pathogens. In a study by Zou et al. [61], bacteria produced several volatile substances with antifungal activity, including acetamide, benzaldehyde, benzothiazole, 1-butanamine, methanamine, and phenylacetaldehyde.

Indirect interactions involve competition, namely the intrinsic ability to utilize the nutrients released by the host plant, as well as mechanisms responsible for the induction of host defense responses. Many species of rhizosphere or phyllosphere yeasts compete with *Fusarium* fungi for micronutrients and macronutrients [56]. Siderophores, the iron-chelating compounds with a high affinity for ferric iron, facilitate competition for Fe^2+^ ions in environments with neutral pH where the availability of Fe^2+^ ions is limited [62]. The *A. pullulans* HN6.2 isolate produced only fusigen, a hydroxamate siderophore [62], whereas *B. subtilis* CAS15 isolate produced the catechol siderophore 2,3-dihydroxybenzoate-glycine-threonine trimeric ester bacillibactin [63].

The use of microorganisms to activate immune responses to *Fusarium* pathogens in cereals has been rarely described in the literature. Petti et al. [24] demonstrated that the non-pathogenic bacterial strain of *Pseudomonas fluorescens* MKB158 elicited host defense responses and protected barley against *F.* *culmorum*. The bacterium significantly influenced the accumulation of 1203 transcripts, and primed 74 positive and 14 negative responses to the pathogen. Barley plants inoculated with *P.* *fluorescens* strain MKB158 improved grain filling, lignin deposition and oxidative responses, and lowered the content of protease inhibitors and proteins that play a key role in programmed cell death [24]. Méndez-Gómez et al. [55] demonstrated that *Azospirillum brasilense* cell wall components influenced superoxide (O_2_^(-)^) production in wheat roots. Roberti et al. [23] found that the *Clonostachys rosea* fungus promotes plant growth, lowers peroxidase expression and enhances chitinase isoforms that are putative cell wall disruptors. The analyzed antagonist also induced the expression of PR4 protein which probably plays an important role in plant defense responses against pathogens.

## 4. Trichothecene Degradation by Microorganisms 

### 4.1. Trichothecene-Degrading Bacteria

**Figure 2:**
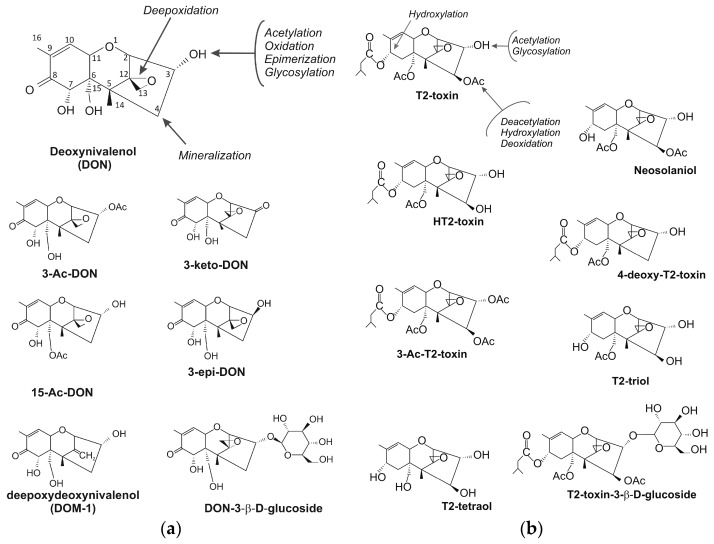
Degradation products of DON (**a**) and T-2 toxin (**b**).

The most common biologically-modified products of DON are DOM-1, 3-epi-DON, and 3-keto DON (Figure 2a). The modification products of T-2 toxin that are most frequently described in literature include HT-2 toxin, T-2 triol, T-2 tetraol, neosolaniol (NEO), 3-acetyl T-2 toxin, and 4-deoxy T-2 toxin (Figure 2b) [64,65,66,67]. Trichothecenes are modified by acetylation, deacetylation, de-epoxidation, epimerization, glucosylation, and oxidation. Trichothecene modification products are generally described as less toxic for humans and animals. In a study by Pierron et al. [26], bacterial de-epoxidation or epimerization of DON altered the interaction between the mycotoxin and the ribosome, which prevented the activation of MAPK (mitogen-activated protein kinase) and reduced toxicity. At the cellular and subcellular level, DON exerts toxic effects by binding to the ribosome, which inhibits the synthesis of proteins and nucleic acids, triggers ribotoxic stress, activates kinases, MAPKs, and their downstream signaling pathways [34]. The products of DON’s microbiological modification do not activate this pathway, which eliminates symptoms of acute or chronic poisoning in humans and animals. Mycotoxin glucosides in plants are referred to as masked mycotoxins because they are not always detected with the use of the existing analytical methods and may be hydrolyzed during digestion in animals or humans [11]. Analytical methods based on liquid chromatography coupled with mass spectrometry (LC-MS) are widely used for the determination of mycotoxins and their plant metabolites. Many plant biotransformation products of mycotoxins are difficult to detect by conventional analytical methods, and no regulatory limits have been set for them in food commodities [68]. Trichothecene glucosides have been isolated from *Fusarium* infected plant material [69]. The group of masked mycotoxins comprises not only some specific mycotoxins, such as DON and zearalenone, but also other *Fusarium* mycotoxins [70].

DON degradation is observed in aerobic and anaerobic organisms. Aerobic bacteria degrade the mycotoxin by oxidation to 3-keto DON with isomerization to 3-epi DON. Anaerobic bacteria [71,72] and aerobic bacteria [73] decompose DON by de-epoxidation to DOM-1 (Table 2). Soil from arable fields, water, cereal leaves and spikes can act as reservoirs of bacterial strains with the above properties. Bacteria isolated from soil where *Fusarium*-infected cereals were grown can rapidly degrade DON, but only on selected media and within a broad range of temperatures (12–40 °C) and pH (6.0–7.5), which suggests that those microorganisms could be applied in cereal production in temperate and warmer climates [73].

Bacteria of the genera *Nocardioides* and *Devosia* can degrade DON to 3-epi-DON and 3-keto DON [74]. After seven days of incubation, *Nocardioides* bacterial strains isolated from wheat rhizosphere degraded 90% of DON to 3-epi-DON and two unidentified compounds in seven days [66]. In a study by Sato et al. [74], 13 tested bacterial isolates of the genera *Nocardioides* and *Devosia* degraded DON at a concentration of 100 μg mL^–1^ to concentrations below the limit of detection (0.5 µg mL^–1^). According to the cited authors, the application of the tested isolates led to the production of various metabolites, which points to the cooperative catabolism of DON in the environment: the biodegradation products of one isolate were degraded by another isolate. According to Ito et al. [75], bacteria of the genus *Marmoricola* obtained from wheat spikes sprayed with DON or inoculated with *F. graminearum* effectively reduced DON contamination levels in wheat and barley grain. The cited studies demonstrated for the first time that bacteria of the genus *Marmoricola* are capable of degrading DON. They also revealed that *Marmoricola* strains can be effectively used to reduce DON levels in feeds containing wheat and barley grain. Cserháti et al. [25] reported that 32 isolates of *Rhodococcus* bacteria were capable of degrading a broad range of mycotoxins, including trichothecenes. The mechanism that was implicated, but not proven, in the degradation process was the high metabolic activity of bacteria containing large nucleoids and linear megaplasmids. These structures encode for oxidases and other enzymes which enable bacteria to compete for energy and carbon derived from organic compounds. In the above study, the concentrations of T-2 toxin were reduced already 24 h after the bacterial suspension had been introduced to the substrate. The trichothecene-degrading potential of bacteria from cropped soil can be utilized to protect crops or introduce genetic modifications to cereals to increase their resistance to pathogens of the genus *Fusarium.* The introduction of Biomin^®^ BBSH 797 for detoxifying trichothecenes in animal feed was a commercial success on the EU market. This product contains bacterial strain *Eubacterium* BBSH 797 which is isolated from bovine rumen fluid [76] and has the ability to degrade several trichothecenes [77].

In recent years, bacterial consortia have been used to transform *Fusarium* mycotoxins. Ahad et al. [81] demonstrated that a bacterial consortium containing low levels of Gram-positive anaerobic bacteria, including *Alkaliphilus crotonatoxidans, Bacillus* spp. and *Blautia coccoides*, the aerobic species of *Stenotrophomonas* (Gram-negative), was highly effective in de-epoxidizing eleven trichothecene mycotoxins. They suggested that the soil-derived bacterial consortium contained more than 30% of unidentified bacterial species. The bacterial consortium was characterized by a rapid and stable ability to de-epoxidize DON after 48 h of incubation. Vanhoutte et al. [82] developed a biological test involving *Lemna minor* L. which is eliminated under exposure to DON concentrations higher than 1 mg L^−1^. When microorganisms derived from soil and activated sludge were incorporated into a macronutrient solution for plant growth, DON concentrations of 5 and 50 mg L^−1^ were effectively neutralized and modified. In addition, the metabolites 3-epi-DON and the epimer of de-epoxy-DON (3-epi-DOM-1) were found as biotransformation products of both consortia.

### 4.2. Fungi Capable of Degradation and Acetylation of Trichothecenes 

Trichothecenes are toxic for eukaryotic fungal cells, therefore, fungi producing those mycotoxins have developed mechanisms that protect them against the harmful effects of trichothecenes [67]. In the cells of *Fusarium* pathogens, trichothecenes are biosynthesized by intermediate products of C-3 acetylation, where acetylation is controlled by *TRI101* and *TRI201* genes encoding trichothecene 3-O-acetyltransferase [35,83]. Acetylation of C-3 carbon (DON ⇒ 3-Ac-DON) reduces DON toxicity [84]. Under exposure to trichothecenes, the activity of trichothecene 3-O-acetyltransferase was also reported in the cells of fungi of the genera *Aspergillus*, *Saccharomyces*, *Blastobotrys*, and *Trichomonascus* which do not produce those compounds [65,83].

Yeasts belonging to the *Trichomonascus* clade can degrade T-2 toxin in three ways: by acetylating C-3 carbon, by hydrolyzing the isovaleric group, or by glycosylating C-3 carbon [35]. All three biotransformation products—3-acetyl T-2 toxin, NEO and T-2 toxin glucoside—are significantly less toxic than T-2 toxin [35]. Interestingly, in the group of the 23 analyzed species of yeasts capable of converting T-2 toxin to 3-acetyl T-2 toxin, only three species of *Blastobotrys muscicola*, *B. robertii*, *B. peoriensis* converted T-2 toxin to T-2 toxin 3-glucoside through glycosylation, whereas *Trichomonascus petatosporus* and four species of the genus *Blastobotrys* transformed T-2 toxin to two compounds: 3-acetyl T-2 toxin and NEO [35].

Filamentous fungi such as *Alternaria alternata*, *Rhizopus microsporus* var. *rhizopodiformis* and *Aspergillus oryzae* metabolized DON into hydrolyzable conjugated DON [79]. He et al. [71] demonstrated that *Aspergillus tubingensis* (strain NJA-1) was capable of hydrolyzing DON, but the metabolites participating in this process and the underlying mechanism of action have not been identified.

Kosawang et al. [21] studied the hyperparasitic fungus *Clonostachys rosea* IK726, which is resistant to DON, and demonstrated that the fungus produces several synergistic proteins to activate a number of defense mechanisms against the toxin. A more detailed study revealed that DON induces a transcriptome representing genes that encode metabolic enzymes in *C. rosea* cells, including cytochrome P450 oxidase, cytochrome c oxidase, and stress proteins. The ThiJ/PfpI protein family was most strongly induced in the group of proteins activated by DON. Genes encoding other stress proteins were also expressed under the influence of DON [21]. A recent study by Tian et al. [80] demonstrated that fungi of the genus *Trichoderma* have the same defense mechanisms as plants when exposed to *F. graminearum*, where DON is degraded to D3G.

## 5. Conclusions

Trichothecenes are the most ubiquitous mycotoxins in cereal grains and grain products. *Fusarium* pathogens are difficult to control with the use of chemical agents during the growing season. Organically-farmed cereals are characterized by a more varied microbiome, which contributes to the elimination of *Fusarium* fungi. The application of selected antagonistic microorganisms to cereal crops in the growing season can inhibit the proliferation of *Fusarium* fungi by way of hyperparasitism, competition or antibiosis. Such treatments reduce the severity of fungal infections in grains and indirectly protect plants against infections. Isolates of antagonistic microorganisms effectively reduce trichothecene concentrations in cereal grains by degrading them to non-toxic products or by binding to antagonistic cells. New mycotoxin detection methods and advanced techniques for selecting mycotoxin-degrading microorganisms have led to the development of a commercial product for the biodegradation of trichothecenes in grains and feeds.

## Figures and Tables

**Table 1:** Mechanisms of action by which bacteria and fungi degrade *Fusarium* pathogens.

Microorganisms	Microbial Mechanisms of Action	Pathogens	Reduction in DON Content	Reference
Bacteria				
*Bacillus megaterium*, *Bacillus subtilis*	Not studied	*Fusarium graminearum*	50–89%	[15]
*Bacillus subtilis*	Fungicidal or fungistatic metabolites: surfactin, iturin and fengycin lipopeptides	*F. graminearum*	not studied	[46]
*Bacillus subtilis*	Antifungal lipopeptides, fengycin-like isoforms	*F. graminearum*	51%	[50]
*Bacillus amyloliquefaciens*	Production of antibiotics or competition for nutrients	*F. graminearum*	16–90%	[51]
*Azotobacter nigricans*	Antifungal activity	*F. sporotrichioides*, *F. poae*, *F. crookwellense*, *F. equiseti*, *F. graminearum*, *F. sambucinum*, *F. culmorum*	not studied	[52]
*Bacillus, Pseudomonas*, *Microbacterium*	Promotion of plant growth	*F. culmorum*	not studied	[53]
*Bacillus cereus*	Volatile antagonistic compounds	*F. graminearum*	not studied	[54]
*Pseudomonas fluorescens*	Elicit host defense responses	*F. culmorum*	not studied	[24]
*Azospirillium brasilense*	Elicit host defense responses	root pathogens	not studied	[55]
Yeasts				
*Cryptococcus flavescens*	Competitive inhibition via aggressive colonization of plant	*F. graminearum*	15–18%	[20]
*Metschnikowia pulcherrima*, *Hanseniaspora* sp., *Cyberlindnera sargentensis*, *Aureobasidium pullulans*, *Candida subhashii*, *Pichia kluyveri*	Strong competition for micronutrients and/or macronutrients	*F. poae*, *F. langsethiae*, *F. graminearum*, *F. culmorum*, *F. crookwellense*, *F. oxysporum*	not studied	[56]
*Sphaerodes mycoparasitica*	Hyperparasitism	*F. graminearum*	not studied	[57]
*Trichoderma harzianum*	Antagonistic activity	*F. graminearum*	not studied	[58]
*Trichoderma atroviride*	Hyperparasitism	*F. graminearum*	not studied	[45]
*Clonostachys rosea*	Cell wall degrading enzymes: chitinases, glucanases and proteases	*F. culmorum*	not studied	[59]
*Trichoderma harzianum*	Hyperparasitism elicit host defense responses	*F. culmorum*	not studied	[60]
*Clonostachys rosea*		*F. culmorum*	not studied	[23]

DON-deoxynivalenol.

**Table 2:** Trichothecene-degrading microorganisms.

Microorganisms	Origin of Isolates	Mycotoxins	Efficiency of Biodegradation	Biodegradation Products	Reference
Bacteria					
*Lactobacillus plantarum* and other lactic acid bacteria	Culture collection	DON, T-2 toxin	28–35%	Physical adsorption	[61]
*Nocardioides* sp.	Soil	DON	90%	3-epi-DON	[66]
*Marmoricola* sp.	Wheat spikes	DON	100%	Not studied	[75]
*Serratia*, *Clostridium*, *Citrobacter*, *Enterococcus*, *Stenotrophomonas*, *Streptomyces*	Soil	DON	100%	DOM-1	[73]
*Rhodococcus erythropolis*, *Rh. coprophilus*, *Rh. rhodochrous*, *Rh. globerulus*	Soil/Soil contaminated with oil	T-2 toxin	90%	Not studied	[25]
*Pseudomonas* sp., *Blastobacter* sp., *Arthrobacter* sp., *Rhizobiaceae*	Leaves, soil, water	T-2 toxin	100%	NEO, T-2 tetraol, T-2 triol	[65]
*Curtobacterium* sp.	No data	T-2 toxin	100%	T-2 triol	[78]
*Devosia* sp., *Nocardioides* sp.	Soil, wheat leaves	DON	100%	3-epi DON and other unidentified compounds	[74]
*Bacillus, Anaerofilum*, *Collinsella*, *Clostridiales*	Chicken intestines	DON	32–100%	Not studied	[72]
*Eubacterium* sp.	Rumen	DON		DOM-1	[76]
Yeasts					
*Blastobotrys muscicola, B. robertii*, *B. peoriensis*	Culture collection	T-2 toxin		T-2 toxin 3-β-d-glucoside	[35]
*B. capitulata*, *B. adeninivorans*, *B. mokoenaii*, *B. malaysiensis*, *B. raffinofermentas*, *Trichomonascus petasosporum*, *B. indianensis*			48–100%	NEO	
*T. petasosporun*, *T. ciferri*, *B. indianensis*, *B. adeninivorans*, *B. raffinofermentas*, *B. mokoenaii*, *B. malaysiensis*, *B. nivea*, *B. terestis*, *B. arbuscula*, *B. attinorum*, *B. parvus*, *B. serpentis*, *B. illinoisensis*				3-acetyl T-2 toxin	
Filamentous fungi					
*Alternaria alternata*	Culture collection	DON	91–97%	DON glutathione conjugates	[79]
*Rhizopus microsporus* var. *rhizopodiformis*			93–96%		
*Aspergillus oryzae*			84–97%		
*Aspergillus tubingenesis*	Soil	DON	94%	Hydrolysis	[71]
*Trichoderma harzianum*, *T. koningii*, *T. longibranchiatum*, *T. atroviride*, *T. asperellum*, *T. virens*	Culture collection	DON	70–90%	D3G	[80]

DON: deoxynivalenol, D3G: deoxynivalenol-3-β-d-glucopyranoside, NIV: nivalenol, 3-Ac-DON: 3-acetyl-deoxynivalenol, 15-Ac-DON: 15-acetyldeoxynivalenol, DOM-1: deepoxydeoxynivalenol, NEO: neosolaniol.

## Abbreviations

The following abbreviations are used in this manuscript:

15-Ac-DON	15-Acetyl-deoxynivalenol
3-Ac-DON	3-Acetyl-deoxynivalenol
D3G	Deoxynivalenol-3-β-d-glucopyranoside
DOM-1	De-epoxy-deoxynivalenol
DON	Deoxynivalenol
FHB	*Fusarium* head blight
FPP	Farnesyl pyrophosphate
MAPK	Mitogen-activated protein kinase
NEO	Neosolaniol
NIV	Nivalenol
